# Eight weeks of treatment with mineralocorticoid receptor blockade does not alter vascular function in individuals with and without type 2 diabetes

**DOI:** 10.14814/phy2.16010

**Published:** 2024-04-12

**Authors:** Stine H. Finsen, Mie R. Hansen, Pernille B. L. Hansen, Stefan P. Mortensen

**Affiliations:** ^1^ Department of Cardiovascular and Renal Research, Institute of Molecular Medicine University of Southern Denmark Odense Denmark; ^2^ Department of Nephrology Odense University Hospital Odense Denmark

**Keywords:** aldosterone, endothelial function, mineralocorticoid receptor, type 2 diabetes

## Abstract

Aldosterone has been suggested to be involved in the microvascular complications observed in type 2 diabetes. We aimed to investigate the effect of mineralocorticoid receptor (MR) blockade on endothelial function in individuals with type 2 diabetes compared to healthy controls. We included 12 participants with type 2 diabetes and 14 controls. We measured leg hemodynamics at baseline and during femoral arterial infusion of acetylcholine and sodium nitroprusside before and 8 weeks into treatment with MR blockade (eplerenone). Acetylcholine infusion was repeated with concomitant n‐acetylcysteine (antioxidant) infusion. No difference in leg blood flow or vascular conductance was detected before or after the treatment with MR blockade in both groups and there was no difference between groups. Infusion of n‐acetylcysteine increased baseline blood flow and vascular conductance, but did not change the vascular response to acetylcholine before or after treatment with MR blockade. Skeletal muscle eNOS content was unaltered by MR blockade and no difference between groups was detected. In conclusion, we found no effect of MR blockade endothelial function in individuals with and without type 2 diabetes. As the individuals with type 2 diabetes did not have vascular dysfunction, these results might not apply to individuals with vascular dysfunction.

## Graphical abstract

Visual summary of the current study.

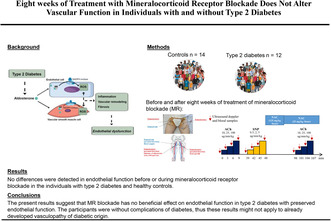

## 1 INTRODUCTION

The prevalence of type 2 diabetes is increasing worldwide (Kenny & Abel, 2019; Petrie et al., 2018). Individuals with type 2 diabetes are known to have a higher rate of co‐morbidities and cardiovascular events (Bruder‐Nascimento et al., 2014; Garg et al., 2015; O'Keefe et al., 2008), and aldosterone has been linked as a potential mediator (Luther, 2016), contributing to the development of cardiovascular complications (Bender et al., 2013; Kenny & Abel, 2019). Aldosterone exerts its effects on endothelial cells and vascular smooth muscle cells (VSMC) in the vascular wall, through a slow genomic pathway by induction of nicotinamide adenine dinucleotide phosphate (NADPH) oxidase, in addition to an rapid non‐genomic response through endothelial nitric oxide synthase (eNOS) activation (Förstermann & Li, 2011; Montezano & Touyz, 2012). In endothelial cells, aldosterone increases the formation of nitric oxide (NO), possibly through the non‐genomic pathway, whereas aldosterone‐induced NADPH oxidase activation could generate reactive oxygen spices (ROS) in VSMC as well as promote increased Ca^2+^ influx, inducing vasoconstriction. In the healthy vasculature, the overall effect of aldosterone appears to be vasodilation (Furchgott & Zawadzki, 1980; Palmer et al., 1987; Skøtt et al., 2006). In contrast, infusion of physiological levels of aldosterone causes vasoconstriction in individuals with type 2 diabetes (Finsen et al., 2020). This vasoconstrictor response was attenuated after infusion of the antioxidant n‐acetylcysteine, suggesting that NO is involved in the altered vascular response to aldosterone in type 2 diabetes.

In conditions with elevated aldosterone levels, mineralocorticoid receptor (MR) over activation can reduce NO bioavailability and synthesis due to increased eNOS uncoupling and NADPH oxidase activity. This might lead to impairment of the NO‐dependent vasodilation of the vessels, in addition to a possible increase in NADPH oxidase activity, thus contributing to further generation of ROS (Barrera‐Chimal & Jaisser, 2019; Schafer et al., 2010; Skøtt et al., 2006). The rapid vasoconstrictor response of aldosterone signaling in VSMC, augments the pathological effects of the MR in the vessels in addition to a diminished NO generation, which could facilitate pathological effects of the MR (Kolkhof & Bärfacker, 2017). Even minor elevations of aldosterone, within the physiological levels, have previously been shown to contribute to an increased risk of cardiovascular mortality, within the population of type 2 diabetes (Bender et al., 2013; Lyngsø et al., 2016). In addition, epidemiological studies have demonstrated a clear correlation between elevated plasma aldosterone levels and the risk of cardiovascular disease (Bender et al., 2013).

A previous study in rodents, induced with type 2 diabetes, demonstrated a prevention of vascular remodeling by pharmacological treatment with the MR blockade eplerenone, which was associated with a reduction in vascular oxidative stress (Silva Marcondes Alves et al., 2015). Current therapeutic options to reduce the cardiovascular risk in individuals with type 2 diabetes mainly include blockade of the renin‐angiotensin‐aldosterone system (RAAS) (Bender et al., 2013; McCurley & Jaffe, 2012), in addition to the now known cardiovascular benefits of treatment with sodium–glucose co‐transporter 2 inhibitors (Barrera‐Chimal et al., 2022; Woo, 2020). Adding a MR antagonist to an optimized inhibition of RAAS, has previous been studied. Two major trials, RALES and EPHESUS (Pitt et al., 1999, 2003) found evidence for the beneficial effects of treatment with the non‐selective MR antagonist spironolactone and the selective eplerenone, respectively, on the cardiovascular system in heart patients (Chrysant & Chrysant, 2014; Pitt et al., 1999, 2003; Ziff et al., 2016). Angiotensin II is one of the main mediators for aldosterone production, but treatment with a RAAS inhibitor does not block synthesis of aldosterone completely (Ivanes et al., 2012; Queisser & Schupp, 2012). This leads to aldosterone release despite treatment, which is also known as ‘aldosterone breakthrough’ (Ivanes et al., 2012; Queisser & Schupp, 2012). Reduced coronary flow reserve as an indicator of coronary microvascular dysfunction in type 2 diabetes, in subjects without known clinical ischemic heart disease has previously been investigated. Addition of spironolactone to an optimized treatment with RAAS inhibition, improved coronary flow reserve suggesting excess MR activation (Garg et al., 2015). The additional beneficial effects of blockade of the MR could be a contribution of a further suppression of the effects of aldosterone, thus reducing the ‘aldosterone breakthrough’ (O'Keefe et al., 2008).

The two well‐known pharmacological antagonists of the MR are eplerenone and spironolactone (Struthers et al., 2008). Eplerenone shows the similar efficacy as spironolactone, but is more selective towards the MR even though, it is 20‐fold less potent to the MR compared to spironolactone (Coty et al., 2006; Hughes & Cassagnol, 2020). In addition, eplerenone has a lower cross‐reactivity with androgen and progesterone receptors, compared to Spironolactone, resulting in fewer side effects (Coty et al., 2006; Hughes & Cassagnol, 2020). A post hoc analysis from the EPHESUS trial, showed an absolute risk reduction in cardiovascular events on the subgroup of individuals with type 2 diabetes, when treated with eplerenone (O'Keefe et al., 2008). In addition, the novel non‐steroidal, selective MR antagonist finerenone has shown a reduction of the risk of cardiovascular events in individuals with type 2 diabetes and chronic kidney disease compared with placebo (Agarwal et al., 2022; Barrera‐Chimal et al., 2022). In the FIDELIO‐DKD and FIGARO‐DKD trials, finerenone was added to an optimized RAAS inhibition, showing improved cardiovascular outcomes and a reduced progression of chronic kidney disease in individuals with type 2 diabetes and various stages of chronic kidney disease, compared to placebo (Filippatos et al., 2021, 2022). Although these studies have found cardioprotective effects of MR blockers, it is unclear if this effect is associated to changes in endothelial function as it was not evaluated in these studies.

In vivo animal studies, support that aldosterone contributes to vascular remodeling and endothelial dysfunction, and that these effects are reversed by MR blockade (Schafer et al., 2010; Silva Marcondes Alves et al., 2015). In this study, we therefore aimed to investigate the effect of 8 weeks of mono‐treatment with an aldosterone receptor antagonist (MR blockade) on endothelial function, tissue perfusion and ROS signaling in individuals with type 2 diabetes compared to healthy controls. To address this, we determined leg hemodynamics and endothelial function during intraarterial infusions of an endothelium dependent and non‐dependent vasodilator, both before and during antioxidant infusion. After an 8‐week period of MR blockade, the measurements were repeated. We hypothesized that MR blockade would improve endothelial function in type 2 diabetes.

## 2 MATERIALS AND METHODS

### 2.1 Participants

**FIGURE 1:**
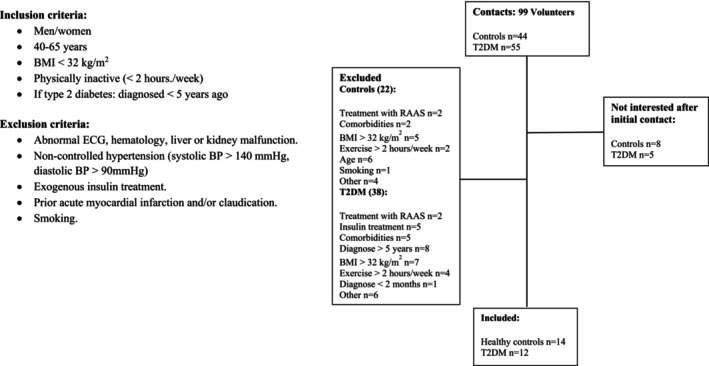
Flow‐chart of included participants. Number (*n*) of individuals who were potentially qualified to participate in the study and number of participants included/excluded in the study. T2D, type 2 diabetes; CON, controls the present data was collected as part of a larger study (Finsen et al., 2021).

Twelve individuals (male/female Caucasians; 7/5), diagnosed with type 2 diabetes <5 years and 14 healthy controls (male/female Caucasians; 5/9) were enrolled in the study (Figure 1). The present data were collected as part of a larger study (Finsen et al., 2021). Participants were recruited from Odense University Hospital patient database (patients with type 2 diabetes), by advertisements in local newspapers and through the internet on pages dedicated to recruitment (individuals with type 2 diabetes and control participants). Individuals with hypertension (>140/90 mmHg), BMI > 32 kg/m^2^, who performed exercise more than 2 h/week, were smokers or in treatment with a renin‐angiotensin‐aldosterone blocker, were excluded. Individuals with type 2 diabetes with complications related to their diabetes (retinopathy, neuropathy and/or nephropathy) were also excluded. The participants did not change their medication following enrollment. All of the participants completed a pre‐experimental day consisting of a resting 12‐lead ECG (MAC800 GE HealthCare, Milwaukee, WI, USA), blood pressure measurement (Omron Healthcare Co., Kyoto, Japan) and a fasting blood screening (glucose, HbA1c, lipids, and markers of hematology, thyroid, liver and kidney function). None of the participants were diagnosed with any cardiovascular disease or demonstrated evidence of liver or renal disease (eGFR <60 mL/min/1.73 m^2^). Baseline characteristics are summarized in Table 1.

**TABLE 1:** Participant characteristics.

Variable	Controls		Type 2 diabetes		*p*	*p*	*p*
	Pre	Post	Pre	Post	Pre CON versus T2D	Post CON versus T2D	Pre versus post
Participants (*n*)	14	14	12	10			
Male/female (*n*)	5/9	5/9	7/5	7/3			
Age	53 ± 9		55 ± 6				
Time since diagnosis	‐		2.4 ± 1.2		0.43		
Weight (kg)	77.3 ± 13.7	77.6 ± 13.6	91.5 ± 7.1	91.4 ± 6.2	0.001	0.003	
BMI (kg/m^2^)	26 ± 3	26 ± 3	30 ± 2	30 ± 2	<0.001	0.001	
Total cholesterol	5.1 ± 0.8	4.9 ± 0.8	4.0 ± 0.5	3.6 ± 0.5	0.003	<0.001	0.06^a^/<0.001^b^
HDL cholesterol	1.6 ± 0.4	1.5 ± 0.3	1.3 ± 0.3	1.3 ± 0.3	0.03	0.07	0.90^a^/0.001^b^
LDL cholesterol	3.0 ± 0.7	2.9 ± 0.8	2.0 ± 0.4	1.7 ± 0.5	<0.001	<0.001	0.09^a^/0.01^b^
Triglycerides	1.12 ± 0.6	1.08 ± 0.5	1.37 ± 0.41	1.33 ± 0.62	0.20	0.24	0.95^a^/0.27^b^
HbA1c (mmol/mol)	35 ± 4	35 ± 4	53 ± 13	51 ± 11	<0.001	<0.001	0.14^a^/0.57^b^
HbA1c (%)	5.3 ± 0.4	5.4 ± 0.4	7.0 ± 1.2	6.8 ± 1.0	<0.001	<0.001	0.17^a^/0.51^b^
Plasma glucose, (mean; mmol/L)	5.9 ± 0.6	5.9 ± 0.6	8.5 ± 1.9	8.3 ± 1.5	<0.001	<0.001	0.34^a^/0.54^b^
Potassium (mmol/L)	4.0 ± 0.3	4.0 ± 0.3	4.2 ± 0.4	4.3 ± 0.5	0.16	0.07	0.92^a^/0.39^b^
Creatinine (μmol L^−1^)	77 ± 16	76 ± 14	75 ± 16	73 ± 20	0.78	0.49	0.75^a^/0.43^b^
Carbamide (mmol/L)	4.7 ± 0.9	5.4 ± 2.0	4.9 ± 1.7	5.3 ± 1.4	0.72	0.90	0.60^a^/0.14^b^
eGFR (mL/min/1.73 m^2^)	81 ± 9	83 ± 9	84 ± 9	85 ± 9	0.44	0.63	0.42^a^/0.83^b^
Systolic blood pressure (mmHg)	127 ± 10	120 ± 11	130 ± 11	127 ± 9	0.50	0.04	0.01^a^/0.09^b^
Diastolic blood pressure (mmHg)	82 ± 9	79 ± 9	84 ± 6	83 ± 7	0.56	0.15	0.05^a^/0.74^b^
Glucose lowering medication (*n*)							
Diet	‐		1				
Metformin	‐		9				
Liraglutid	‐		1				
SGLT2‐inhibitor	‐		1				
Lipid lowering medication (*n*)							
Simvastatin			5				
Atorvastatin	1		3				

*Note*: Values are expressed as mean ± SD or *n* where stated. Pre, prior mineralocorticoid receptor blockade; Post, following mineralocorticoid receptor blockade; CON, controls; T2D, type 2 diabetes; eGFR, estimated glomerular filtration rate; SGLT2‐inhibitor, selective sodium‐glucose cotransporter 2 inhibitor.

^a^ *p*‐value in the control group.

^b^ *p*‐value in the T2D group.

### 2.2 Experimental design

The participants completed an experimental day before (pre) and at the end (post) of 8 weeks of treatment with MR blockade (eplerenone, Teva Denmark A/S, Kgs. Lyngby, Denmark). 24 h prior to each experimental day, all participants refrained from caffeine, alcohol, and exercise. The premenopausal women were tested at the same time point during their menstrual cycle, before and after the intervention period with MR blockade.

**FIGURE 2:**
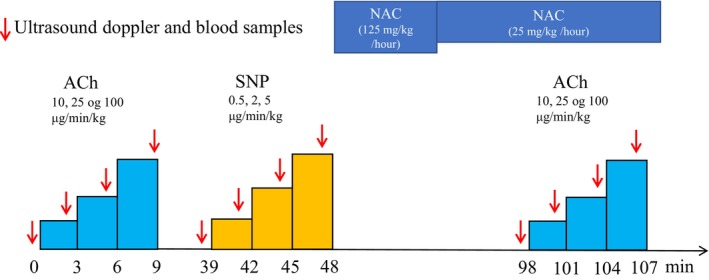
Experimental protocol. Arrows indicate when ultrasound doppler was used to determine steady state conditions, before venous and arterial blood samples were drawn simultaneously. ACh, acetylcholine; SNP, sodium nitroprusside; NAC, n‐acetylcysteine.

On each experimental day, the participants arrived at 08.30 a.m. at the laboratory after an overnight fast (≥8 h) and rested in the supine position for the entire trial. Under aseptic conditions and local anesthesia (Xylocaine 10 mg/mL; AstraZeneca, Mölndal, Sweden), three catheters (18GA; Arrow International Incorporated, Reading, PA, USA) were placed by ultrasound guidance, at a level just proximal for the bifurcation of the common femoral artery, using the Seldinger technique (Seldinger, 1953). A catheter was placed in the femoral artery (pharmacological infusions) and vein (blood samples) of the experimental leg and one catheter was placed in the femoral artery of the non‐experimental leg (blood sampling and blood pressure measurements). Following 20 min of rest, a muscle biopsy was obtained from m. vastus lateralis of the non‐experimental leg using the modified Bergström technique with suction (Bergstrom, 1975). After 20 min of additional rest, the participants completed the following trial (Figure 2): (1) Acetylcholine (ACh) infusion (Miochol‐E; Bausch + Lomb Incorporated, Berlin, Germany): three stepwise 3 min infusions at 10, 25 and 100 μg min^−1^ [kg leg volume]^−1^; (2) Sodium nitroprusside infusion (SNP: Nitropress; Hospira Incorporated, Lake Forest, IL, USA): three stepwise 3 min infusions at 0.5, 2 and 5 μg min^−1^ [kg leg volume]^−1^; (3) N‐acetylcysteine (NAC; antioxidant) infusion (Amgros I/S, Copenhagen, Denmark): 20 min at 125 mg kg^−1^ h^−1^ (loading dose) and subsequently at 25 mg kg^−1^ h^−1^ (maintenance dose), for the rest of the experimental protocol; to potentiate NO bioavailability (Brown et al., 2004); (4) After 50 min of NAC infusion, ACh infusion was repeated. The ACh and SNP trials were separated by 30 min.

### 2.3 Mineralocorticoid receptor blockade

Eplerenone (Teva Denmark A/S, Kgs. Lyngby, Denmark) was used as MR blockade. Following the pre‐experimental day, all participants were assigned to receive 25 mg Eplerenone per day for the initial 2 weeks of the intervention period, followed by an increase to 50 mg per day up to and including the last experimental day. All participants had a blood screen within the first week after initiation, and within 1 week after any change of dose, as hyperkaliemia and elevated azotemia are known adverse effects of eplerenone. Due to the blood pressure lowering effect of eplerenone, each participant performed home blood pressure measurements (Omron Healthcare Co., Kyoto, Japan). To detect possible adverse effects, all participants were contacted at least once a week during the 8‐week intervention period. One subject reported symptoms (migraine), when the dose was increased from 25 mg per day to 50 mg per day and therefore remained on 25 mg per day.

### 2.4 Measurements and calculations

Anthropometric measurements were used for calculation of leg volume (Katch & Katch, 1979). Femoral arterial blood flow (leg blood flow; LBF) was measured by Ultrasound Doppler (Logic E9, GE Healthcare, Pittsburgh, PA, USA), as previously described (Nyberg et al., 2012), the sonographer being blinded towards group assignment. Mean arterial pressure (MAP) and heart rate (HR) were monitored with transducers positioned at the level of the heart (pressure monitoring kit, ref. T450217A; Edwards Lifesciences Corporation, Irvine, CA, USA). Recordings were made via a data acquisition system (PowerLab 16/30; ADInstruments, Bella Vista, NSW, Australia), for later software analysis (Labchart 8; ADInstruments, Bella Vista, NSW, Australia). MAP (mmHg) was calculated over 8–16 cycles from the area under the arterial pressure curve. Leg vascular conductance (LVC) was calculated as LBF/MAP. Arterial and venous blood samples were drawn simultaneously before and during the individual infusion trials (2 min). Venous EDTA‐blood was obtained and centrifuged <15 min (4°C) at 4000 rpm for 10 min. Plasma aliquots were frozen at −80°C.

### 2.5 Analysis

Plasma aliquots were analyzed for stable metabolites of nitrite and nitrate ([NO*x*]; NO_2_^−^ + NO_3_^−^) measured by a commercially available colorimetric assay kit (Cayman Chemical Co., RRID: Cat# 780001, Nitrate/Nitrite Colorimetric Assay Kit, Ann Harbor, MI, USA). Plasma was diluted 1:2 and analyzed in duplicates following the instructions of the manufacturer.

### 2.6 Western immunoblotting

The muscle biopsies were instantly frozen in liquid nitrogen and stored in −80°C freezer awaiting western immunoblotting (WB). Western immunoblotting was performed on biopsies before and after 8 weeks of mineralocorticoid blockade. Protein was extracted from each muscle biopsy with RIPA Lysis Buffer (10×; EMD Millipore Corporation, Billerica MA, USA) and complete Tablets, Mini (cat.04693124001, Roche Diagnostics GmbH, Mannheim, Germany) was added the lysisbuffer. The Protein lysate was mixed with NuPAGE Sample Reducing Agent (cat.NP0009, 10×; Thermo Fisher Scientific, Invitrogen, Carlsbad, USA), NuPAGE LDS Sample Buffer cat.NP0007 (4×; Thermo Fisher Scientific, Invitrogen, Carlsbad, USA), and heat denatured. Samples were run on Criterion™ TGX Stain‐Free Precast Gels 4%–15% cat.5678084 (Bio‐Rad Laboratories, Incorporated, USA), and subsequently blotted onto a Midi format PVDF 0,2 μm Trans‐Blot Turbo Transfer membrane (cat.1704157, Bio‐Rad Laboratories, Incorporated, CA, USA). Membranes was blocked in 5% milk in Tris‐Buffered Saline with 0,1% Tween20 (TBST) and then presented with the following primary antibodies, Purified Mouse Anti‐eNOS/NOS Type III, cat.610297 (BD Biosciences, BD Transduction Laboratories™, USA) and Rabbit polyclonal to GAPDH—Loading Control (cat.ab9485, Abcam plc., Cambridge, UK). For secondary antibodies, Polyclonal Goat anti‐Mouse Immunoglobulins/HRP (cat.P0447, Dako Denmark Aps., Agilent Technologies Denmark Aps, Glostrup, DK) and Polyclonal Goat Anti‐Rabbit Immunoglobulins/HRP (cat.P0448; Dako, Agilent Technologies Denmark Aps, Glostrup, Denmark) were used. All antibodies were diluted in TBST. Blots were developed using Western Lighting enhanced chemiluminescence (ECL) pro (cat.NEL122001EA; PerkinElmer Incorporated, Massachusetts, USA) For anti‐eNOS/NOS Type III the enhancer SuperSignal West Femto Maximum Sensitivity Substrate (cat.34095; Thermo Fisher Scientific, Carlsbad, USA) was supplemented (1:4) to the ECL mix.

### 2.7 Statistical analysis

An unpaired t‐test with Welch's correction was used in comparison of between group baseline characteristics. Prior to analysis, a D'Agostino & Pearson test was conducted to verify the normality assumption, and log‐transformation conducted if necessary. Outcome variables (LBF, LVC, Leg O_2_, [NOx] and eNOS expression) were determined, using one‐way repeated measures ANOVA followed by Tukey's honestly significant difference post hoc test or, if inevitable, by nonparametric analysis followed by Dunn's multiple comparison post hoc test. Between groups, type 2 diabetes and healthy controls, the outcome variables were determined, using a two‐way repeated measure ANOVA. A mixed model two‐way repeated measures ANOVA was used in case of missing data. Pairwise differences were identified, using the Sidak post hoc procedure. *p* < 0.05 was considered significant. All data are presented as mean ± SD. Statistical analysis was conducted by GraphPad Prism (GraphPad Prism version 8.4.3 for Windows, GraphPad Software, San Diego, California USA, www.graphpad.com).

Due to technical difficulties, all data could not be obtained during parts of the trials, either pre or post intervention (*n* = 10; controls: *n* = 7, type 2 diabetes: *n* = 3).

## 3 RESULTS

### 3.1 Participant characteristics

The individuals with type 2 diabetes had a higher BMI and weight compared to the controls both before (BMI: *p <* 0.001, weight: *p* = 0.001) and during (BMI: *p* = 0.001, weight: *p <* 0.001) MR blockade. HbA1c and plasma glucose were higher in the individuals with type 2 diabetes, both before (*p <* 0.001) and at the end of (*p <* 0.001) the intervention with MR blockade. Prior to treatment with MR blockade, total cholesterol, LDL and HDL cholesterol were lower in the individuals with type 2 diabetes compared to the control group. Systolic blood pressure was lowered by MR blockade in the control group (*p* = 0.01), tended to be lowered in the individuals with type 2 diabetes (*p* = 0.09), and was higher in the individuals with type 2 diabetes compared to the control group (*p* = 0.04). MR blockade did not alter diastolic blood pressure in either group. There was no change in variables of kidney function or potassium after MR blockade in either group and there was no difference between the groups (Table 1).

### 3.2 Leg hemodynamics and plasma [NOx] levels in response to infusion of acetylcholine before and during MR blockade

**FIGURE 3:**
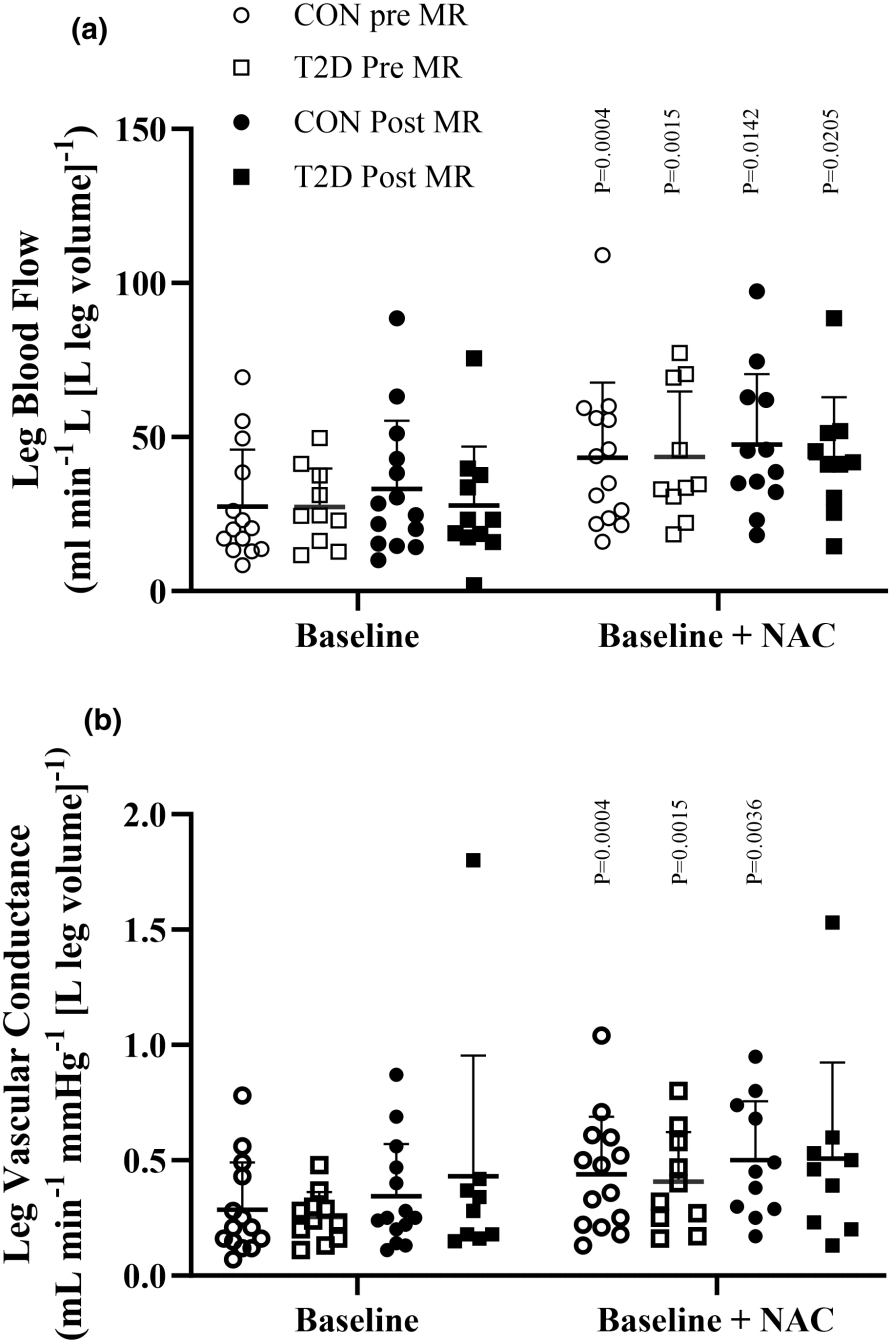
Baseline values of leg blood flow and leg vascular conductance. Baseline values of leg blood flow (LBF; panel a a) and leg vascular conductance (LVC; panel b) compared to baseline values concomitant with n‐acetylcysteine (NAC: 20 min at 125 mg∙kg^−1^ h^−1^ (loading dose), subsequently 25 mg∙kg^−1^ h^−1^ (maintenance dose) before (and during mineralocorticoid receptor (MR) blockade. Following MR blockade, baseline LBF and LVC increased in the control group during co‐infusion with NAC, compared to infusion of ACh alone, but not in the T2D group compared to baseline. Data were analyzed by two‐way repeated measures ANOVA and presented as mean ± SD. T2D, type 2 diabetes participants; CON, control participants. *p*‐values indicate difference from baseline within the group.

**FIGURE 4:**
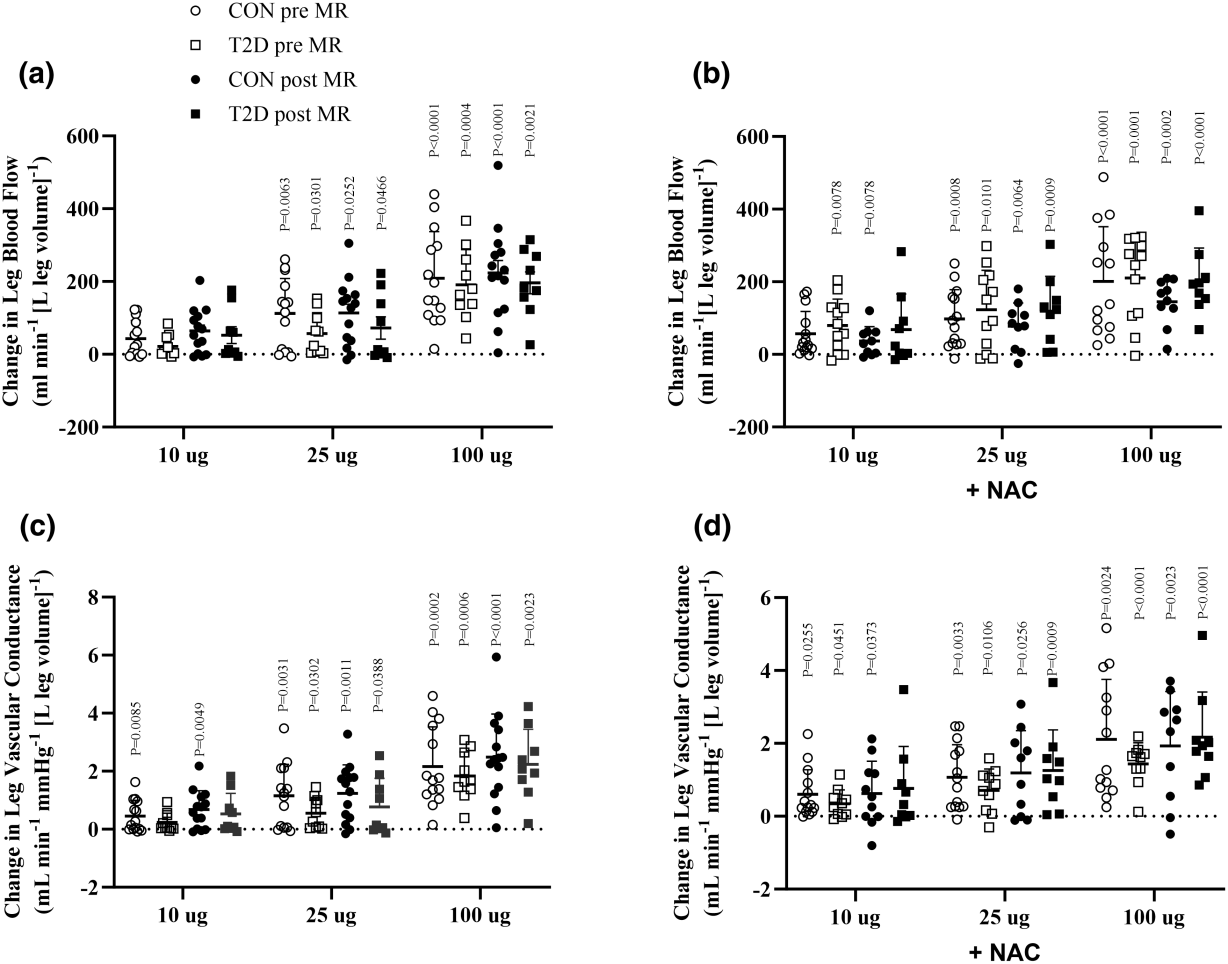
Leg blood flow and leg vascular conductance during acetylcholine infusion. Leg blood flow during (LBF; panel a + c) and leg vascular conductance (LVC; panel b + d) during incremental doses of infused acetylcholine (ACh; 0.5, 2, and 5 μg min^−1^ [kg leg volume]^−1^) with and without co‐infusion with n‐acetylcysteine (NAC) before (T2D, *n* = 12 and CON, *n* = 14) and during (T2D, *n* = 10 and CON, *n* = 14) mineralocorticoid receptor (MR) blockade. Data were analyzed by one and two‐way repeated measures ANOVA and presented as mean ± SD. T2D, type 2 diabetes participants; CON, control participants; Pre, before MR blockade; Post, after MR blockade. *p*‐values indicate difference from baseline within the group.

**FIGURE 5:**
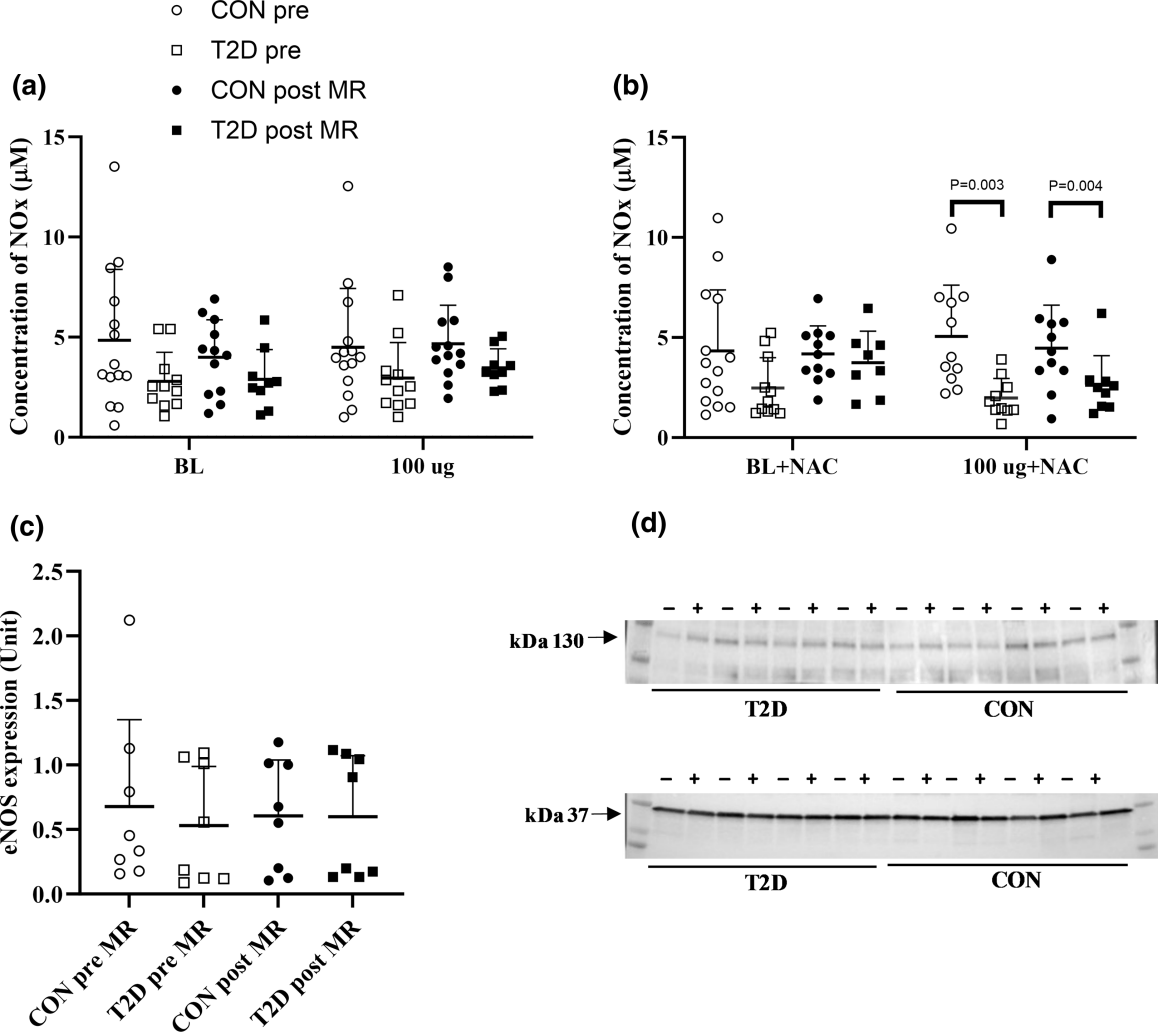
Venous levels of plasma [NOx]. Venous levels of plasma [NOx] during infusion of acetylcholine without (panel a) and with concomitant infusion of n‐acetylcysteine (NAC; 20 min at 125 mg∙kg^−1^ h^−1^ (loading dose), subsequently 25 mg∙kg^−1^ h^−1^ (maintenance dose): panel b) before (T2D, *n* = 12 and CON, *n* = 14) and during (T2D, *n* = 10 and CON, *n* = 14) 8‐weeks of mineralocorticoid (MR) blockade in individuals with and without type 2 diabetes. Skeletal muscle eNOS expression (panel c and d) Bands consistent with an equal expression of eNOS (~130 kDa) both before and after MR blockade (before indicated by a − and after indicated by a +;T2D, *n* = 8, CON, *n* = 8). GAPDH (~37 kDa) was used as loading control. Full western immunoblotting and are shown in Online Resource Figure S2 (https://figshare.com/s/9de47d72868f322bc61d). Data were analyzed by two‐way repeated measures ANOVA and data are presented as mean ± SD. ACh: acetylcholine, NAC, n‐acetylcysteine; T2D, type 2 diabetes participants; CON, control participants; [NO], level of plasma nitric oxide concentration; Pre, before MR blockade; Post, during MR blockade. *p*‐values indicate difference between groups.

At baseline, there was no difference in LBF or vascular conductance between the individuals with type 2 diabetes and the control group (Figure 3; Table S1). During infusion of ACh, LBF and vascular conductance increased in both groups, and there was no difference between groups (Figure 4). ACh infusion did not alter [NOx] in either group and there was no difference between groups (Figure 5).

Infusion of NAC increased baseline LBF and vascular conductance in both groups, but there was no difference in the ACh‐induced increase in LBF and vascular conductance, compared to ACh alone in either group. During infusion at the high dose of ACh, plasma [NOx] was lower in the individuals with type 2 diabetes compared to the control group (type 2 diabetes: 1.98 (1.35, 3.18) μmol L^−1^ vs. controls: 5.05 (2.39, 7.04) μmol L^−1^; *p* = 0.003). Compared to baseline, MAP was lower in the control group during infusion of ACh at 10 and 25 μg min^−1^ [kg leg volume]^−1^, while no change occurred in MAP in the individuals with type 2 diabetes (Figure S1).

After 8 weeks of MR blockade, there was no difference in baseline LBF or vascular conductance, and no difference in the ACh‐induced increase in LBF and vascular conductance compared to before MR blockade, and no difference between the individuals with type 2 diabetes and the control group. Plasma [NOx] remained unchanged in both groups during ACh infusion, and there was no difference between groups. No difference in MAP was detected during infusion of ACh, between the individuals with type 2 diabetes and control group, compared to prior MR blockade.

Infusion with NAC, increased baseline LBF and vascular conductance in the control group, whereas only LBF was increased in the individuals with type 2 diabetes. No difference was detected between groups. During the high dose of ACh infusion in the presence of NAC, plasma [NOx] was lower in the individuals with type 2 diabetes compared to the control group (type 2 diabetes: 2.62 (1.53, 2.88) μmol L^−1^ vs. controls: 4.47 (2.13, 5.94) μmol L^−1^; *p* = 0.04). No difference in plasma [NOx] were detected in the individual groups, compared to prior to MR blockade. There was no difference in MAP during ACh infusion between the individuals with type 2 diabetes and the control group and no difference compared to prior to MR blockade.

### 3.3 Leg hemodynamics during sodium nitroprusside infusion before and after MR blockade

**FIGURE 6:**
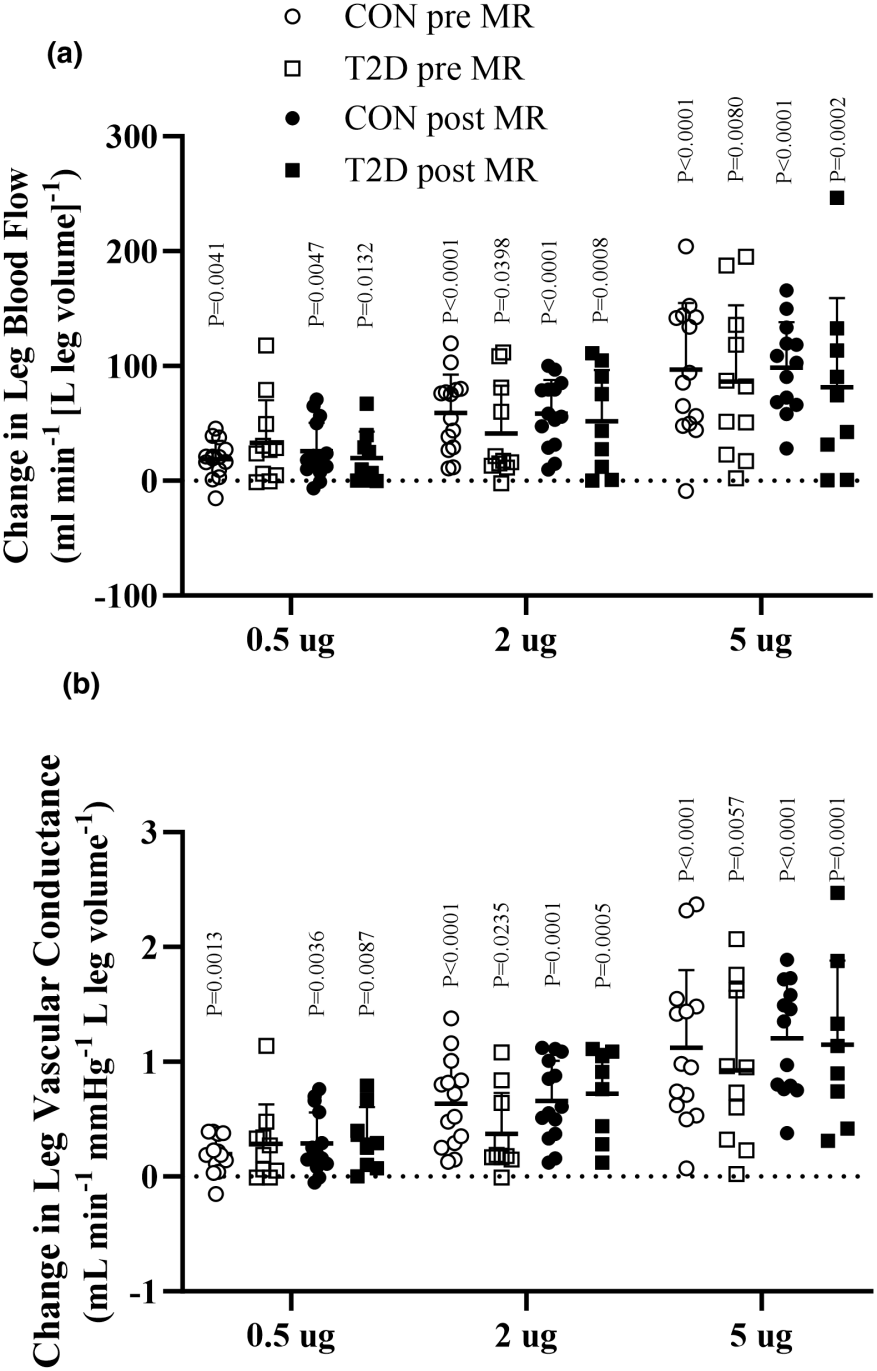
Leg blood flow and leg vascular conductance during sodium nitroprusside infusion. Change in leg blood flow during (LBF; panel a) and leg vascular conductance (LVC; panel b) during incremental doses of infused sodium nitroprusside compared to baseline (SNP; 0.5, 2, and 5 μg min^−1^ [kg leg volume]^−1^) before (T2D, *n* = 12 and CON, *n* = 14) and during (T2D, *n* = 10 and CON, *n* = 14) mineralocorticoid blockade (MR blockade). Data were analyzed by one and two‐way repeated measures ANOVA and presented as mean ± SD. T2D, type 2 diabetes participants; CON, control participants; Pre, before MR blockade; Post, during MR blockade. *p*‐values indicate difference from baseline within the group.

During SNP, a dose‐dependent increase in LBF and vascular conductance, was detected in the control group, and an increase occurred in LBF and vascular conductance, in the individuals with type 2 diabetes, at 2 μg and 5 μg·min^−1^ [L leg volume]^−1^ compared to baseline (Figure 6; Table S2). MAP decreased during SNP infusion, in both the individuals with type 2 diabetes and the control group (Figure S2), with no difference between groups.

Following MR blockade, a dose‐dependent increase in LBF and vascular conductance, was present in the individuals with type 2 diabetes, compared to baseline, similarly to the control group. There was no difference in the SNP‐induced change in LBF and vascular conductance, between the individuals with type 2 diabetes and control group (Table S3).

### 3.4 Expression of eNOS before and after MR blockade

Western immunoblotting analysis of human skeletal muscle showed equal migrated bands at ~130 kDa with similar intensity in all samples both before and after 8 weeks of mineralocorticoid blockade, indicating no difference in the expression of eNOS between the individuals with type 2 diabetes and the control group, or in the individual groups before or after MR blockade (Figure 5c,d; Figure S3).

## 4 DISCUSSION

In this study, we investigated the vascular response to 8 weeks of MR blockade, in individuals with type 2 diabetes compared to healthy controls, and the effect of MR blockade on NO bioavailability and the expression of eNOS. We hypothesized that MR blockade would improve the endothelial function in individuals with type 2 diabetes. Both before and after 8 weeks of MR blockade, we observed no difference in endothelial function in the individuals with type 2 diabetes compared to the control group, or within the individual groups. This could indicate that MR blockade does not alter endothelial function in these populations absent endothelial dysfunction.

### 4.1 Vascular function prior to MR blockade

Prior to initiation of MR blockade, there was no difference in LBF or vascular conductance, between the individuals with type 2 diabetes and the control group, during infusion of incremental doses of ACh and SNP. This was finding was unexpected, as type 2 diabetes has been associated to endothelial dysfunction (Huynh et al., 2014; Tooke, 1995). The similar vascular response to infused ACh in the two groups is supported by the similar skeletal muscle eNOS content and similar plasma [NOx] in both groups during infusion of Ach as the ACh‐mediated vasodilation has been demonstrated to be mainly NO‐dependent (Calles‐Escandon & Cipolla, 2001; Mortensen et al., 2009; Natali & Ferrannini, 2012). A possible explanation for the lack of difference in endothelial function between the individuals with type 2 diabetes and the control group is that none of the included participants had any previous medical history regarding micro‐ or macro‐vascular complications. This might in part be based on the included individuals with type 2 diabetes were all diagnosed <5 years, and thus were early in their course for their disease.

Infusion of NAC increased baseline LBF and vascular conductance in both the individuals with type 2 diabetes and the control group, prior to MR blockade, indicating increased bioavailability of NO (Jeremias et al., 2009; Nogueira et al., 2018; Sun, 2010). During infusion of ACh in the presence of NAC, the vascular conductance increased in a dose‐dependent manner compared to baseline in the individuals with type 2 diabetes, which was comparable to the response in the control group.

### 4.2 Vascular function at the end of 8 weeks MR blockade

Following MR blockade, LBF or vascular conductance was similar between individuals with type 2 diabetes and controls during infusion of ACh or SNP. This is in contrast to a previous study demonstrating that MR blockade improves coronary flow reserve, an indicator of endothelial function, within the type 2 diabetes population (Garg et al., 2015). However, the individuals with type 2 diabetes did not have endothelial dysfunction prior to MR blockade in the present study and this is likely to explain the difference in findings.

Despite no difference in baseline values of LBF in the individuals with type 2 diabetes, after MR blockade, an increase in LBF still occurred during the high dose of infused ACh in the presence of NAC compared to baseline during solely NAC infusion. A lower plasma [NOx] was observed in the individuals with type 2 diabetes compared to the control group, similar to prior initiation with MR blockade. Similarly, an equal expression of eNOS was identified by western immunoblotting between the individuals with type 2 diabetes and the control group, as well as in the individual groups. This emphasizes that no effect of MR blockade on endothelial function appeared in the individuals with type 2 diabetes.

MR blockade in diabetes has previously been examined in the FIDELIO‐DKD trial, where a cardiovascular benefit was noted in the finerenone group independently of pre‐existing cardiovascular disease (Filippatos et al., 2021). In addition, in the FIGARO‐DKD trial, finerenone reduced the cardiovascular mortality and morbidity in the time to the first occurrence of cardiovascular event in individuals with type 2 diabetes compared to placebo (Barrera‐Chimal et al., 2022). In contrast to the present results, the included participants in both the FIDELIO‐DKD and the FIGARO‐DKD trials had established cardiovascular disease, which was not present in the included participants presented in this study. In both the FIDELIO‐DKD and the FIGARO‐DKD trials, finerenone was added to an optimized regimen of RAAS inhibition (Filippatos et al., 2021, 2022). In the present study, MR blockade was used as a mono‐therapy.

A major limitation to the present study was that the included individuals with type 2 diabetes did not have endothelial dysfunction when compared to the control group. Another limitation was that eight of the twelve included participants in the type 2 diabetes group were in treatment with statins. In addition to statins lipid‐lowering properties, lower levels of plasma aldosterone (Andersson & Vasan, 2015; Baudrand et al., 2015) and antioxidant properties decreasing ROS generation has been demonstrated (Drapala et al., 2014). However, careful evaluation of the individual results suggests that there was no difference in the response between the individuals with type 2 diabetes with and without treatment with statins. The absence of initial endothelial dysfunction in the individuals with type 2 diabetes due to statins treatment therefore seems unlikely. 50% of the included individuals with type 2 diabetes presented with a BMI between 30 and 32 kg [m^2^]^−1^ and were thus considered obese. Careful evaluation of the individual results suggested that there was no difference in the response between individuals with BMI <30 and ≥30 kg [m^2^]^−1^, and the lack of beneficial effect of the MR blockade, due to obesity, are therefore considered unlikely. An inclusion criterion was a sedentary lifestyle with exercise <2 h a week and the performed exercise was not to be high‐intensity training. This assumption was based on the individual subjects own reporting, but not recorded.

## 5 CONCLUSIONS

We found no effect of 8 weeks of blockade of the aldosterone sensitive MR on endothelial function in participants with and without type 2 diabetes. None of the enrolled subjects had any known cardiovascular disease, and the individuals with type 2 diabetes did not have any medical history with micro‐ or macro‐vascular complications. Therefore, these results might not apply to individuals with already developed vasculopathy of diabetic origin. MR blockade has been shown to improve coronary flow reserve in individuals with type 2 diabetes, reducing their risk of cardiovascular disease (Garg et al., 2015), especially when added to an already optimized blockade of the RAAS (Barrera‐Chimal et al., 2022). Yet, it is still undetermined if MR antagonists has a place in the treatment of individuals with type 2 diabetes without already known vascular complications.

## AUTHOR CONTRIBUTIONS

Stine H. Finsen collected the data, analyzed, and interpreted the data and drafted the manuscript. Mie R. Hansen collected the data, analyzed, and interpreted the data and revised the manuscript. Pernille B. L. Hansen was responsible for conception and design, analyzed and interpreted the data and revised the manuscript. Stefan P. Mortensen was responsible for conception and design, collecting the data, analyzing and interpretation of the data and drafting the manuscript. All authors approved of the final version of the manuscript.

## FUNDING INFORMATION

This work was supported by grants from the Independent Research Fund Denmark (6110–00187), The Region of Southern Denmark's Research Pool (16/13529) and the Danish Medical Association Research fund (2638669).

## CONFLICT OF INTEREST STATEMENT

Stine H. Finsen, Mie R. Hansen and Stefan P. Mortensen declare they have no financial interests. Pernille B. L. Hansen is full time employee and Executive Director, head of Bioscience Renal, AstraZeneca, Göteborg, Sweden. The company has not been involved in the design of the study, data collection or analysis of the data. Xylocaine used for local anesthesia is produced by AstraZeneca, but the company has not provided anything for the study free of charge. The authors declare no conflicts of interests.

## ETHICS STATEMENT

The study protocol was reviewed and approved by the Ethics Committee of Copenhagen and Region of Southern Denmark (H‐15007940) and registered at ClinicalTrials.gov (NCT03017703). The project was conducted in accordance with the *Declaration of Helsinki*. All participants were informed both verbally and in writing before informed consent was obtained. The authors affirm that all participants provided informed consent for publication of the collected data.

## CLINICAL TRIAL REGISTRATION

NCT03017703. https://clinicaltrials.gov/ct2/show/NCT03017703.

## Supporting information


Data S1.

## Data Availability

Readers are alerted to the fact that additional materials related to this manuscript may be found at https://figshare.com/s/9de47d72868f322bc61d. The datasets generated during the present study are not publicly available, due to local legislation but are partly available from the corresponding author on reasonable request.
