# Supplementary material for: Eight weeks of treatment with mineralocorticoid receptor blockade does not alter vascular function in individuals with and without type 2 diabetes
Source: Physiol Rep. 2024 Apr 12;12(7):e16010. doi: 10.14814/phy2.16010 (PMC11014871; doi:10.14814/phy2.16010)
Supplement: Supplementary file 1 — Data S1. [file PHY2-12-e16010-s001.docx]

| Infusion of Acetylcholine | | | | | | | |
| --- | --- | --- | --- | --- | --- | --- | --- |
| Baseline | | **10 μg** | | **25 μg** | | **100 μg** | |
| CON | **T2D** | **CON** | **T2D** | **CON** | **T2D** | **CON** | **T2D** |
| Leg blood flow before MR blockade (ml min^-1^ [L leg volume]^-1^) | | | | | | | |
| 27±18 | 27±13 | 70±18 | 49±37 | 140±101* | 84±62* | 237±126* | 218±101* |
| Leg blood flow after MR blockade (ml min^-1^ [L leg volume]^-1^) | | | | | | | |
| 33±22 | 30±18 | 97±73 | 76±82 | 147±98* | 95±105* | 257±138* | 207±117* |
| Leg vascular conductance before MR blockade (mL min^-1^ mmHg-1 [L leg volume]^-1^) | | | | | | | |
| 0.3±0.2 | 0.3±0.1 | 0.7±0.6* | 0.5±0.4 | 1.4±1.1* | 0.8±0.6* | 2.5±1.4* | 1.9±1.0* |
| Leg vascular conductance after MR blockade (mL min^-1^ mmHg-1 [L leg volume]^-1^) | | | | | | | |
| 0.3±0.2 | 0.3±0.2 | 1.0±0.8* | 0.8±0.9 | 1.6±1.1* | 1.0±1.1* | 3.0±1.6* | 2.3±1.5* |

**Online Table 1. Leg blood flow and leg vascular conductance during infusion of acetylcholine.** Leg blood flow and leg vascular conductance incremental doses (10, 25 and 100 μg min^−1^ [kg leg volume]^-^1) of infused acetylcholine (ACh) before and after mineralocorticoid blockade (MR blockade). Data are presented as mean ± SD. T2D: type 2 diabetes participants. CON: control participants. **p* < 0.05 in the individual group compared to baseline. No difference was noted between groups.

| Infusion of Acetylcholine + N-acetylcysteine | | | | | | | |
| --- | --- | --- | --- | --- | --- | --- | --- |
| Baseline | | **10 μg + NAC** | | **25 μg + NAC** | | **100 μg + NAC** | |
| CON | **T2D** | **CON** | **T2D** | **CON** | **T2D** | **CON** | **T2D** |
| Leg blood flow before MR blockade (ml min^-1^ [L leg volume]^-1^) | | | | | | | |
| 43±24 | 44±21 | 100±71 | 80±46* | 141±89* | 121±65* | 245±159* | 189±61* |
| Leg blood flow after MR blockade (ml min^-1^ [L leg volume]^-1^) | | | | | | | |
| 48±23 | 43±21 | 127±90* | 112±117 | 170±123* | 162±114* | 258±129* | 244±108* |
| Leg vascular conductance before MR blockade(mL min^-1^ mmHg-1 [L leg volume]^-1^) | | | | | | | |
| 0.4±0.1 | 0.4±0.2 | 1.0±0.2* | 0.8±0.4* | 1.5±0.3* | 1.1±0.6* | 2.5±0.5* | 1.8±0.6* |
| Leg vascular conductance after MR blockade(mL min^-1^ mmHg-1 [L leg volume]^-1^) | | | | | | | |
| 0.5±0.3 | 0.4±0.3 | 1.2±1.0* | 1.2±1.4 | 1.6±1.2* | 1.7±1.3* | 2.6±1.4* | 2.6±1.4* |

**Online Table 2. Leg blood flow and leg vascular conductance during infusion of acetylcholine concomitant with n-acetylcysteine (NAC)**. Leg blood flow and leg vascular conductance during incremental doses (10, 25 and 100 μg min^−1^ [kg leg volume]^-^1) of infused acetylcholine concomitant with n-acetylcysteine (ACh+NAC) before and after mineralocorticoid blockade (MR blockade). Data are presented as mean ± SD. T2D: type 2 diabetes participants. CON: control participants. NAC: n-acetylcysteine. **p* < 0.05 in the individual group compared to baseline. No difference was noted between groups.

**Online Figure 1 Measurement of mean arterial pressure during acetylcholine**

Mean arterial pressure during infusion of acetylcholine (ACh; 10, 25 and 100 μg min^−1^ [kg leg volume]^-^1) (panel a) and ACh co-infused with n-acetylcysteine (NAC) (panel b) prior to and following MR blockade. Data were analysed by a mixed model one-way ANOVA followed by Sidak multiple comparisons post hoc test within groups and presented as mean ± SD. NAC: n-acetylcysteine. CON: control participants. T2D: type 2 diabetes participants. Pre: prior to MR blockade. Post: following MR blockade. **p* < 0.05 compared to baseline within the individual group. No difference was detected between groups.

**Online Figure 2 Measurement of mean arterial pressure during sodium nitroprusside**

Mean arterial pressure during infusion with sodium nitroprusside (SNP; 0.5, 2 and 5 μg min^−1^ [kg leg volume]^-1^) prior to and following mineralocorticoid blockade (MR blockade). Data were analysed by a mixed model one-way ANOVA followed by Sidak multiple comparisons post hoc test within groups and presented as mean ± SD. CON: control participants. T2D: type 2 diabetes participants. Pre: prior to MR blockade. Post: following MR blockade. **p* < 0.05 vs. baseline in the individual group. No difference was detected between groups.

| Infusion of Sodium nitroprusside | | | | | | | | | | |
| --- | --- | --- | --- | --- | --- | --- | --- | --- | --- | --- |
| Baseline | | **0.5 μg** | | | **2 μg** | | | **5 μg** | | |
| CON | **T2D** | | **CON** | **T2D** | | **CON** | **T2D** | | **CON** | **T2D** |
| Leg blood flow before MR blockade (ml min^-1^ [L leg volume]^-1^) | | | | | | | | | | |
| 31±21 | 30±11 | | 49±21* | 59±38 | | 90±41* | 64±41* | | 127±58* | 106±65* |
| Leg blood flow after MR blockade (ml min^-1^ [L leg volume]^-1^) | | | | | | | | | | |
| 33±25 | 38 ±30 | | 59±29* | 70±54* | | 91±30* | 110±60* | | 132±47* | 153±93* |
| Leg vascular conductance before MR blockade (mL min^-1^ mmHg-1 [L leg volume]^-1^) | | | | | | | | | | |
| 0.3±0.2 | 0.3±0.1 | | 0.5±0.2* | 0.6±0.4 | | 0.9±0.5* | 0.7±0.4* | | 1.4±0.7* | 1.2±0.7* |
| Leg vascular conductance after MR blockade (mL min^-1^ mmHg-1 [L leg volume]^-1^) | | | | | | | | | | |
| 0.3±0.2 | 0.4±0.3 | | 0.6±0.3* | 0.7±0.6* | | 1.0±0.3* | 1.1±0.8* | | 1.5 ±0.6* | 2.0±1.2* |

**Online Table 3. Leg blood flow and leg vascular conductance during infusion of sodium nitroprusside.** Leg blood flow and leg vascular conductance during incremental doses (0.5, 2 and 5 μg min^−1^ [kg leg volume]^-1^) of infused sodium nitroprusside (SNP) before and after mineralocorticoid blockade (MR blockade). Data are presented as mean ± SD. T2D: type 2 diabetes participants. CON: control participants. **p* < 0.05 in the individual group compared to baseline. No difference was noted between groups.

**Gel 1 anti eNOS/NOS III 1:200**

**b**

**a**

**̶ + ̶ + ̶ + ̶ + ̶ + ̶ + ̶ + ̶ +**

**Gel 2 anti eNOS/NOS III 1:200**

**kDa**

**130**


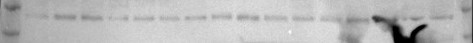


**̶ + ̶ + ̶ + ̶ + ̶ + ̶ + ̶ + ̶ +**

**kDa**

**130**


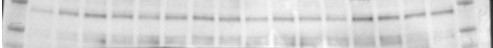


**T2D**

**CON**

**CON**

**T2D**

**c**

**Gel 2 anti GAPDH 1:5000**

**d**

**̶ + ̶ + ̶ + ̶ + ̶ + ̶ + ̶ + ̶ +**

**Gel 1 anti GAPDH 1:5000**

**kDa**

**37**

**kDa**

**37**


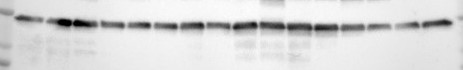


**̶ + ̶ + ̶ + ̶ + ̶ + ̶ + ̶ + ̶ +**


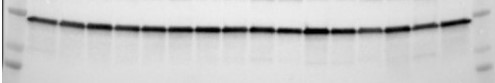


**CON**

**T2D**

**CON**

**T2D**

**Online Figure 3 Western immunoblotting identifying expression of eNOS.** Western immunoblotting identifying expression of eNOS in human skeletal muscle, before and after eight weeks of mineralocorticoid receptor (MR) blockade. Equal migrating bands at ~130 kDa with similar intensity in all samples both before and after eight weeks of mineralocorticoid blockade, indicating no difference in the expression of eNOS between the individuals with type 2 diabetes and the control group, or in the individual groups. Panel a and b are the individual subjects before and after MR blockade; displayed next to one another; before indicated by a ̶ and after indicated by a +. Panel c shows anti GADPH related to a. Panel d shows anti GADPH related to b. T2D: individual with type 2 diabetes. CON: control group.
